# The Local Action of the So-Called Astringents on the Blood-Vessels

**Published:** 1877-01

**Authors:** 


### ARTICLE IX.

### *The Local Action of the so-called Astringents on the Blood-vessels.*

H. Rosenstirn, )*Würzb Phys. Med. Verhandlungen*, ix. p. 32, and *Centralblatt für die Medicin. Wissenschaften*, No. 35) tested solutions of nitrate of silver, acetate of lead, tannic, gallic, and pyrogallic acids, sesquichloride of iron, and alum, by placing them upon the mesentery of a curarized frog, and estimating the size of the lumen of the affected vessel by means of the microscope. Nitrate of silver acted most powerfully on the vessels; it was employed in solution from one to ten per cent. The observation was often interfered with by the torbidity produced in the tissues. The contraction extended to about half the lumen of both arteries and veins, and in a much less degree in the capillaries. The reaction occurred within a few seconds. Stagnation generally took place in the vessels; permanent in the capillaries, and temporary in the arteries and veins. Tannic acid had exactly the opposite effect. Under its influence the arteries dilate, even the veins and capillaries to the extent of one-half of their lumen, and appear to be overfilled with blood corpuscles. The dilated vessels become narrowed at once by the action of nitrate of silver. Gallic and pyrogallic acids have an action similar to tannic acid. Acetate of lead acts more feebly than nitrate of silver. It narrows the arteries and veins, but no effect was observed on the capillaries. Sometimes also stand-still of the circulation occurs. A ten per cent. solution of sesquichloride of iron has no effect; in a fifty per cent. solution it narrowed the vessels less than acetate of lead. This narrowing is limited to the arteries and veins, while the capillaries dilate. Coagulation and discoloration of the blood within the vessels often occur. The results of the alum solution were variable.

In order to exclude a reflex action, the spinal cord was destroyed and the heart ligatured.

The author therefore ascribes an astringent effect, *i. e.* contraction of the vessels, only to nitrate of silver and acetate of lead; whilst this action is doubtful for alum and solution of sesquichloride of iron, and is certainly not the case with the group to which tannic acid belongs.—*London Med. Record.*

